# Does "Flattening the Curve" Affect Critical Care Services Delivery for COVID-19? A Global Health Perspective

**DOI:** 10.34172/ijhpm.2020.117

**Published:** 2020-07-07

**Authors:** Ramiro E. Gilardino

**Affiliations:** HE–Xperts Consulting LLC, Miami, FL, USA.

**Keywords:** COVID-19, Intensive Care Units, Pandemic, SARS-Cov-2, Resource Use

## Abstract

During this coronavirus disease 2019 (COVID-19) global pandemic, nations are taking bold measures to mitigate the spread of Severe acute respiratory syndrome coronavirus 2 (SARS-CoV-2) infections in order to avoid the overwhelming its critical care facilities. While these "flattening the curve" initiatives are showing signs of impeding the potential surge in COVID-19 cases, it is not known whether these measures alleviate the burden placed on intensive care units. Much has been made of the desperate need for critical care beds and medical supplies, especially personal protective equipment (PPE). But while these initiatives may provide health systems time to bolster their critical care infrastructure, they do little to protect the most essential element – the critical care providers. This article examines bolder initiatives that may be needed to both protect crucial health systems and the essential yet vulnerable providers during this global pandemic.

## Background

Since emerging from Wuhan province in China at the end of 2019, severe acute respiratory syndrome coronavirus 2 (SARS-CoV-2) has quickly encircled the globe causing widespread coronavirus disease 2019 (COVID-19).^1^ According to John Hopkins University, by May 30th of this year, the virus has been detected in 185 countries, infecting 6 million people, resulting in 368 700 deaths. After affecting European countries, it quickly spread to the United States, South America, Russia, and India, concentrating most of the active cases.^2^

Imperial College London models estimate that by the end of March 2020, between 7 and 43 million people across 11 European countries were potentially infected with SARS-CoV-2, accounting for 1.88% to 11.43% of the total population.^3^

While 80% of those infected may be asymptomatic or may be able to manage their mild symptoms at home, the prognosis for the 20% who become seriously ill presents a serious challenge to health systems globally. Roughly 4% to 5% of those who become seriously ill with COVID-19 will require hospitalization. Further, about 28% to 30% of these hospitalized patients will be admitted to the intensive care unit (ICU). With neither effective treatment nor a vaccine available, the management of these patients remains solely with the life support measures in the ICU, where patients require an average length of stay of 3 weeks. In the first cohort of 41 patients with COVID-19 from Wuhan, China, 13 (31.7%) patients were admitted to an ICU, and 6 (14.6%) died. Subsequent Wuhan data found 11.1% of ICU patients worsened and died.^4^

Acknowledging the high transmissibility of SARS-CoV-2 and the enormous burden COVID-19 places on healthcare systems, the World Health Organization (WHO) declared COVID-19 a global pandemic.^5^

## Flattening the Curve

Since WHO’s pandemic declaration, nations are enacting ‘flatten the curve’ measures to suppress the potential surge of infections that could both cripple our health systems and increase the risk of death for the most vulnerable populations.^6^ From social distancing and school closures to strict lockdowns and confinement orders, these measures tend to reduce the reproduction rate of SARS-CoV-2, suppressing potential peaks in the number of COVID-19 cases into more manageable (although potentially longer) plateaus, thereby relieving the sudden, crippling pressure on healthcare facilities.

Curve flattening measures have been rapidly enacted worldwide. By mid-March, most countries in Europe implemented different measures to contain the spread, as shown in Table 1. On March 13, the US government declared a state of national emergency, leading to a range of social distancing interventions across the country, including closures of schools, bars, cinemas, and restaurants, cancellation of large public gatherings, including cultural and sporting events, and discouraging gatherings of more than 50 people. Later on, several South American countries implemented lockdown measures and by the time of writing this piece, roughly half of the world population is under a confinement strategy designed to flatten the curve.^7,8^

**Table 1:** 2020 Strategies to “Flatten the Curve” in 5 European Countries^a^

	**School Closures** ^b^	**Banning of Mass Gatherings** ^c^	**Self-quarantine** ^d^	**Working FromHome** ^e^	**Closure of Non-essential Services** ^f^	**Lockdown** ^g^	**Average No. of Daily Cases Since First Local Transmission to Locking Down** ^h^	**Average No. of Daily Cases Since Locking Down to April 9**
France	March 14	March 13	March 17	March 17	March 16	March 17	364	4446
Germany	March 14	March 22	March 6	March 12	March 12	-	1082	5235
Italy	March 5	March 9	March 9	March 9	March 9	March 11	656	4522
Spain	March 12	March 14	March 17	March 14	March 14	March 14	249	5235
United Kingdom	March 21	March 16	March 12	March 16	March 24	March 24	539	3950

^a^Countries selected based on cumulative cases by March 31, 2020. Source: Own elaboration based on data from Flaxman et al.^3^ ^b^Formal closure of teaching institution. ^c^Cancelation of events with large amount of people (sports, music, etc). ^d^Home isolation for those symptomatic or close contacts. ^e^Encourage work from home when possible avoiding commuting and workers displacement. ^f^All non-essential businesses (supermarkets, food retailers, pharmacies) are closed. ^g^Movement restriction of people, encourage all stay at home. ^h^ *P* < .0001 on unpaired two-tailed *t* test. Data from WHO report contains a link to the following page: https://www.who.int/emergencies/diseases/novel-coronavirus-2019/situation-reports.

Through these combined strategies, a sustained fall in the reproduction number from 4·5 average to 1.43 has been inferred, potentially avoiding 59 000 deaths in Europe.^3^ Analyzing these measures from a system-thinking perspective, it remains to be seen whether these initiatives are sufficient to alleviate the burden in the ICU.

As described in Table 2, despite the fall in the reproduction rate in Europe, the extreme pressure on critical care services continues, especially in Spain and Italy, where the surge exceeded the installed capacity of ICU beds; followed by France, and the United Kingdom in a minor scale. In the United States, the Institute for Health Metrics and Evaluation at the University of Washington estimated shortages of roughly 8018 ICU beds and 15 000 mechanical ventilators by mid-April 2020.^10^ During the peak, several states like New York were affected by the surge, and several ICU collapsed.^11^ Chile and Peru are facing similarities with Europe during the pandemic peak^12^; In Brazil, where the containment measures were involved in political hurdles, the ICUs are still under extreme pressure.

**Table 2:** Disease Reproduction and Critical Care Resources Consumption in 5 European Countries*

**Country**	**Population** ^a^	**Attack Rate** **Mean % [SD]** ^b^	**Projected No. of Infected/100 000 Inhabitants** ^b^	**Average ICU Beds/100 000 inhabitants** ^c^	**Average No. of Ventilators/100 000 inhabitants** ^c^	**Potential Cumulative No. of Critical Cases/100** **000 Inhabitants**^d^	**Potential ICU Daily Admission/100 000 inhabitants by March 31 With Containment Strategies** ^e,g^	**Potential ICU Daily** **Admission/100 000 Inhabitants by March 31 Without Containment Strategies** ^f,g^
France	66 977 107	3.00 [1.10-7.40]	3000	12	8	7	0.32	1.76
Germany	82 905 782	0.72 [0.28–1.80]	720	29	30	2	0.08	0.81
Italy	60 421 760	9.80 [3.20-26.00]	9800	1	8	22	1.05	6.07
Spain	46 796 540	15.00 [3.70-41.00]	15 000	10	5	34	1.60	2.75
United Kingdom	66 460 344	2.70 [1.20-5.40]	2700	7	12	6	0.29	0.73

Abbreviation: ICU, intensive care unit. ^*^Countries selected based on cumulative cases by March 31, 2020. Source: Own elaboration based on data: ^a^ World Bank OECD Population figures, https://data.worldbank.org/indicator/SP.POP.TOTL?locations=OE&view=chart. ^b^ Assumptions based on Imperial College London model.^3^ ^c^ Based on data published.^9^ ^d^ Assumption based on the worst case scenario of 28% ICU admission rate. ^e^ Potential critical cases divided by 21 days (average length of stay on ICU). ^f^ Projections if no measures implemented based on ICL model.^3^ ^g^ *P* < .0001 on unpaired two-tailed *t* test.

While nations enact transmission-abatement measures that may be sufficient to successfully flatten the curve, most ICUs will remain critically vulnerable to even a modest surge in COVID-19 cases.

## Curve-Flattening Allows Time to Address Infrastructure and Supplies

Faced with a flattened COVID-19 surge, health systems benefit from the critical gift of time – time that may be used to ensure critical resources, such as temporary hospitals, are put in place to alleviate the overflow from their current infrastructure. For example, China built two dedicated hospitals in Wuhan within 10 days, creating capacity for more than 2000 COVID-19 patients. A prolonged plateau could also provide time to increase their reserves of critical medical equipment, especially additional mechanical ventilators and personal protective equipment (PPE).

Building sufficient capacity is by no means easy. The United Kingdom is seeking any domestic manufacturer willing to produce 30 000 ventilators, while Spain and Italy are increasing their ventilator production capacity, and several manufacturers are creating open-source ventilators. Even if production of critical equipment, such as ventilators, is immediate, it remains unclear how long until production will meet demand or whether these devices will meet regulatory requirements to effectively ventilate patients who suffer from impaired lung function due to severe acute respiratory distress syndrome.

## The Most Critical Vulnerability in ICU Is not Infrastructure

Yet the most crucial component of these health systems is not the infrastructure, but rather the human resources. The multitudes of diverse healthcare workers – physicians, nurses, respiratory therapists, pharmacists, mental health professionals, and other allied healthcare professionals who work together to deliver care to these critically ill patients. Perhaps nowhere is this coordination of human resources more critical than in the ICU. Providing care to critically ill patients requires trained multidisciplinary professionals (intensivists) capable of managing this complex disease.

The Task of the American College of Critical Care Medicine and the Society of Critical Care Medicine recommends that all Level 1 ICUs be staffed by an intensivist “round the clock” 24/7^13^ as the presence of an intensivist is associated with a decreased risk of in-hospital mortality.^14^ Today, the COVID-19 pandemic has forced a reimagining of the ICU workforce, putting the intensivist in a coordinating role, overseeing allied professionals, fellows, and residents, guiding them to deliver care outside the ICU. Many nations have taken bold measures to shore up their ICU workforce. Spain and Italy have recalled retired physicians and nurses, while also recruiting foreign doctors who were awaiting licensure in these countries; the United Kingdom and the United States are exploring similar options.^15,16^ Some health systems are deploying medical students to conduct upfront triage, or even graduating medical students early to join the healthcare system to fight COVID battle.^17^

Yet in building the ICU workforce, it is important to note that intensivists are not interchangeable with untrained healthcare professionals and doing so their lacks in technical skills could further endanger the health of extremely vulnerable COVID-19 patients. While expanding the production pipeline for infrastructure and medical supplies is challenging, the opportunity to increase the number of intensivists to levels sufficient to accommodate a surge of global COVID cases is virtually impossible.

This pandemic is attacking an already understaffed critical care system. For at least the last 15 years, the number of intensivists has been decreasing worldwide, leaving most ICUs incapable of providing optimal levels of care.^18^ Moreover, in the United States, roughly a quarter of critical care physicians and pulmonologists are aged 60 and older, putting them at a higher risk of COVID-related complications. And while sufficient PPE is mandatory, healthcare workers worldwide argue that they are not adequately protected due to its shortage.^19^

## Bolder Measures Are Needed to Strengthen Critical Care Services

Lessons can be learned from countries that appear to have contained the COVID-19 surge. China imposed a strict quarantine in mid-January. These quarantine measures were strengthened greatly with additional initiatives, including widespread testing, strict temperature monitoring, effective supply-chain management for critical medical equipment (eg, ventilators, bronchoscopes, hemodialysis machines, ultrasound machines, PPE, and sterilizing equipment),^20^ and dispatching healthcare workers to areas experiencing a surge in cases. Through these measures, China experienced a gradual reduction in cases, achieving no community transmissions for the first time on March 19.

Other Asian countries and territories (Hong Kong SAR, Singapore, and Taiwan) built upon their experience combatting the 2003 SARS virus to manage and suppress the SARS-CoV-2 infection rate. Singapore implemented extensive tracing of contacts from 14 days before the estimated infection date, also testing and isolating all symptomatic contacts.^21^ South Korea successfully managed to reduce its initial surge in SARS-CoV-2 infection rate by using the “test, trace, treat and isolate” strategy, supported by mobile technology and data analytics. Data from cell phones, closed-circuit and bank transactions used to track movements of SARS-CoV-2 infected people and sending around text messages to their close contacts. This approach proved to be both an efficient and low-cost solution to contact tracing.

As infections spread across Europe and into North America, many countries limited testing to symptomatic cases and their close contacts – no active testing before the local transmission phase. Although Germany, the Netherlands, and Norway adopted measures to expand testing beyond symptomatic people to include younger, potentially asymptomatic persons and other at-risk populations, it is unknown whether these expanded testing measures contributed to lower mortality rates reported by these countries. However, it might have relieved the pressure on ICUs in those countries. Now that most European countries are beyond the initial peak, it is important to know what proportion of the population could have been asymptomatic. These data will provide reassurance of the most accurate SARS-CoV-2 transmission rate that can then inform a more flexible social distancing protocol. Close monitoring of the transmission potential of the virus based on reliable and publicly available data in near real-time will be crucial to short-term forecasts and sound public health decisions.^6^

The surge capacity of critical care is another measure to consider: On February 20 of this year, a 39-year-old healthy male was the first case admitted to the ICU of a local hospital in the Lombardy region of Italy with COVID-19. The following four days, 650 additional cases of COVID-19 were reported in the region, and cities across the Veneto, Emilia-Romagna, and Lombardy regions underwent strict lockdown. 48-hour after the first ICU admission, Italy used to manage the surge capacity cohorting ICU among 15 hub hospitals gaining 130 dedicated beds. By March 7, the number of patients hospitalized in the region with COVID-19 totaled 2217 – 6% (n = 359) of whom requiring ICU beds, at that time there were 482 dedicated beds and patients tested negative for COVID-19 were transferred to ICU outside the region through a national coordinating center.^22^

Low- to middle-income countries with inadequate acute care beds and high bed occupancy rates will struggle during this pandemic. For example, ICU capacity in Africa averages 5 ICU beds per 1 million people, compared to the 4000 ICU beds per 1 million people in Europe.^23^ Mitigation strategies to release ICU beds will put pressure on already constrained health systems and could result in a rise in mortality from other medical conditions. In these countries, the capacity building support in terms of human and technical resources are not the only relevant factor. Efforts should be put in strengthening the education of the population, advising on how to early recognize COVID-19 symptoms and how to seek proper medical care. Healthcare workers, including community health workers, awareness should be created, leading to active searching for potential cases. Testing, tracing contacts, and isolating provides an accurate picture of the situation, to upfront the cases during the increasing phase.

## Mirroring the Past, Looking at the Future

As described, no health system can sustain an uncontrolled outbreak without substantially exceeding its total ICU capacity. Stronger containment measures and early case detection are two short term measures that avoid the sudden collapse of the health system.

The examples presented here are trustworthy sources that can support clinicians and decision-makers on allocating critical care resources before or even during the pandemic peak.

Longer-term initiatives may include stronger measures to ensure that critical care services remain viable while also implementing strategies to reduce the shortage of physicians, nurses, and other allied health professionals in the ICU and update and prepare plans to face upcoming diseases with pandemic potential.

## Acknowledgements

The author acknowledges Michele Cleary, for proofreading and writing support and Susan Rifkin for providing comments in the manuscript.

## Ethical issues

Not applicable.

## Competing interests

Author declares that he has no competing interests.

## Author’s contribution

REG is the single author of the paper.

## References

[1] Wu JT, Leung K, Leung GM (2020). Nowcasting and forecasting the potential domestic and international spread of the 2019-nCoV outbreak originating in Wuhan, China: a modelling study. Lancet.

[2] Johns Hopkins University. Johns Hopkins Coronavirus Resource Center. https://coronavirus.jhu.edu/map.html. Accessed April 10, 2020. Published 2020.

[3] Flaxman S, Mishra S, Gandy A (2020). Estimating the effects of non-pharmaceutical interventions on COVID-19 in Europe. Nature.

[4] Du R-H, Liu L-M, Yin W (2020). Hospitalization and Critical Care of 109 Decedents with COVID-19 Pneumonia in Wuhan, China. Ann Am Thorac Soc.

[5] World Health Organization. WHO Director-General’s opening remarks at the media briefing on COVID-19 - 11 March 2020. WHO Director General’s speeches. https://www.who.int/dg/speeches/detail/who-director-general-s-opening-remarks-at-the-media-briefing-on-covid-19---11-march-2020. Published 2020. Accessed April 17, 2020.

[6] Chowell G, Mizumoto K (2020). The COVID-19 pandemic in the USA: what might we expect?. Lancet.

[7] AFP. 3.9 billion people now in Covid-19 confinement. New Strait Times. 2020. https://www.nst.com.my/world/world/2020/04/580873/39-billion-people-now-covid-19-confinement. Accessed April 18, 2020.

[8] Yeyati L, Malamud A. How to Think About the Lockdown Decision in Latin America. Am Q. April 2, 2020. https://www.americasquarterly.org/article/how-to-think-about-the-lockdown-decision-in-latin-america/. Accessed May 11, 2020.

[9] Rhodes A, Ferdinande P, Flaatten H, Guidet B, Metnitz PG, Moreno RP (2012). The variability of critical care bed numbers in Europe. Intensive Care Med.

[10] Institute for Health Metrics and Evaluation. COVID-19 Projections. https://covid19.healthdata.org/united-states-of-america. Accessed April 11, 2020. Published 2020.

[11] Richardson S, Hirsch JS, Narasimhan M (2020). Presenting characteristics, comorbidities, and outcomes among 5700 patients hospitalized with COVID-19 in the New York City area. JAMA.

[12] Vergara Eva. Chile’s hospital ICUs near full capacity as pandemic rages. Medical Press. https://medicalxpress.com/news/2020-05-chile-hospital-icus-full-capacity.html. Published May 27, 2020. Accessed June 28, 2020.

[13] Masud F, Lam TYC, Fatima S (2018). Is 24/7 In-House Intensivist Staffing Necessary in the Intensive Care Unit?. Methodist Debakey Cardiovasc J.

[14] Sakr Y, Moreira CL, Rhodes A (2015). The Impact of Hospital and ICU Organizational Factors on Outcome in Critically Ill Patients. Crit Care Med.

[15] Ruiz-Tagle J, Esteller R. Sanidad llama a médicos y enfermeros jubilados para evitar la saturación en los hospitales - elEconomista.es. elEconomista.es. https://www.eleconomista.es/sanidad/noticias/10417175/03/20/Sanidad-llama-a-medicos-y-enfermeros-jubilidos-para-evitar-la-satucion-en-los-hospitales.html. Published March 15, 2020. Accessed May 11, 2020.

[16] British Medical Association. COVID-19: retired doctors returning to work. https://www.bma.org.uk/advice-and-support/covid-19/returning-to-the-nhs-or-starting-a-new-role/covid-19-retired-doctors-returning-to-work?gclid=CjwKCAjw_-D3BRBIEiwAjVMy7LTy0mHPYXbWhfbtdbZbNltWm2xuYxqr1Q3NG45oj_e3TsedotauUhoC_E8QAvD_BwE. Published 2020. Accessed May 11, 2020.

[17] Goldberg E. Early Graduation Could Send Medical Students to Virus Front Lines. The New York Times. March 23, 2020. https://www.nytimes.com/2020/03/26/health/coronavirus-medical-students-graduation.html. Published March 23, 2020. Accessed May 11, 2020.

[18] Buchman TG, Coopersmith CM, Meissen HW (2017). Innovative Interdisciplinary Strategies to Address the Intensivist Shortage. Crit Care Med.

[19] Ferioli M, Cisternino C, Leo V, Pisani L, Palange P, Nava S (2020). Protecting healthcare workers from SARS-CoV-2 infection: practical indications. Eur Respir Rev.

[20] Liao X, Wang B, Kang Y (2020). Novel coronavirus infection during the 2019–2020 epidemic: preparing intensive care units—the experience in Sichuan Province, China. Intensive Care Med.

[21] Lee VJ, Chiew CJ, Khong WX (2020 March). Interrupting transmission of COVID-19: lessons from containment efforts in Singapore. J Travel Med.

[22] Grasselli G, Pesenti A, Cecconi M (2020). Critical Care Utilization for the COVID-19 Outbreak in Lombardy, Italy. JAMA.

[23] WHO. Regional Office for Africa. COVID-19 pandemic expands reach in Africa. https://www.afro.who.int/news/covid-19-pandemic-expands-reach-africa. Accessed April 10, 2020. Published 2020.

